# Transmission and reduction of aerosols in classrooms using air purifier systems

**DOI:** 10.1063/5.0044046

**Published:** 2021-03-23

**Authors:** Sebastian Burgmann, Uwe Janoske

**Affiliations:** 1 School of Mechanical and Safety Engineering, University Wuppertal, 42097 Wuppertal, Germany; 2 Steinbeis Research Center Flow Analysis, 45239 Essen, Germany

## Abstract

SARS-CoV-2 (COVID-19) as an airborne respiratory disease led to a bunch of open questions: how teaching in classrooms is possible and how the risk of infection can be reduced, e.g., by the use of air purifier systems. In this study, the transmission of aerosols in a classroom is analyzed numerically and experimentally. The aerosol concentration in a classroom equipped with an air purifier system was measured with an aerosol spectrometer (optical particle sizer, TSI Incorporated) at different locations. The transient reduction of the aerosol concentration, which was artificially generated by an aerosol generator (di-ethyl hexyl sebacate-atomizer, detected particle size ranging from 0.3 to 10 *μ*m), was monitored. The experimental results were used to validate a numerical simulation model of the classroom using the Open Source Computational Fluid Dynamics code OpenFOAM® (version 6). With the numerical simulation model, different scenarios with infected persons in the room have been analyzed, showing that the air purifier system leads to a significant reduction of airborne particles in the room dependent on the location of the infected person. The system can support additional ventilation strategies with fresh air, especially in cold seasons.

## I. INTRODUCTION

The SARS-CoV-2 (COVID-19) pandemic led to dramatic changes throughout the world and had a huge impact on teaching activities in classrooms. The most important factor contributing to the rapid growth of COVID-19 infections is the higher viral load of the SARS-CoV-2 virus in the upper respiratory tract and the shedding of virus-laden droplets during normal activities such as talking and breathing (Bai *et al.*^1^). Unfortunately, there is a knowledge gap about the effectiveness of different strategies such as social distancing, wearing of face masks, etc. as pointed out by Asadi *et al.*,^2^ Bourouiba,^3^ Mittal *et al.*,^4^ or Seminara *et al.*^5^ A lot of studies show the influence of respiratory droplets especially on the COVID-19 pandemic (Chaudhuri *et al.*,^6^ Wang *et al.*,^7^ Cummins *et al.*,^8^ and Li *et al.*^9^), the distribution of the virus due to cough and sneeze (Pendar and Páscoa^10^ and Busco *et al.*^11^), as well as face shields and face masks (Verma *et al.*^12^). Discussions about ventilation strategies by opening windows (if possible), the use of face masks during class or office work, and the possible risk of getting infections are numerous. Understanding and controlling of droplet flows and aerosols seems to be the key mechanism to minimize the infection risks as pointed out by Mittal *et al.*,^13^ Seminara *et al.*,^5^ or Kohanski *et al.*^14^

As the airborne transmission of aerosols was identified as one of the most dominating way of getting infected, the transmission and residence time of aerosols in rooms is of utmost interest. The transport of droplets over larger distances is mostly driven by ambient flows. The importance of ventilation in indoor environments like classrooms is well known (Tang *et al.*^15^ and Li *et al.*^16^). Thanks to the power of computational fluid dynamic (CFD) modeling, the airborne transmission of droplets is an excellent method to evaluate infection risks. Most recently, the analysis of the possibility of infections was demonstrated with numerical simulations during the presidential debate in 2020 by Shao and Li.^17^ Therefore, concerning air purifier systems, CFD is a suitable way to get a deeper insight into the possible effectiveness of such a system. A comprehensive overview of modeling methods was given by Vuorinen *et al.*^18^

An overview on the effects on ventilation, in general, was given by Bhagat *et al.*^19^ Most studies available in literature concerning ventilation concepts focus on specific configurations. This includes application of HVAC (Heating, Ventilation and Air Conditioning) systems and open windows. For example, Thatiparti *et al.*^20^ investigated ventilation concepts for an isolation room, Yang *et al.*^21^ focus on an airliner cabin section, and Yu *et al.*^22^ numerically investigated an office room. The simulation-based study of an COVID-19 outbreak in a restaurant was examined by Liu *et al.*,^23^ showing the influence of the air-conditioning. The transmission of aerosols in underground car parking areas by fans was presented by Nazari *et al.*,^24^ identifying critical zones. An analysis of the particle transmission in school buses was presented by Zhang *et al.*^25^ The influence of HVAC systems and open windows was determined with a CFD analysis. Especially concerning the flow in the classroom of a university, a study was demonstrated by Ascione *et al.*^26^ for different ventilation concepts. Abuhegazy *et al.*^27^ showed the influence of glass barriers in ventilated classrooms on the transmission and deposition of particles with different sizes. Most studies mentioned earlier are based on a steady-state flow field; a one-way coupling of particle tracking was used to describe the particle tracks.

It has to be kept in mind that indoor spaces like air cabins, buses, restaurants, offices, classrooms, etc. can have highly complex flows. This is caused not only by ventilation systems leading to recirculatory flows but also by thermally driven flow effects due to humans, machines, electronic devices, etc. (Craven and Settles,^28^ Licina *et al.*,^29^ and Kohanski *et al.*^14^). These flows significantly affect the distribution of droplets and aerosols.

Quantifying infection risks solely based on aerosol flows calculated by CFD is challenging due to uncertain boundary conditions, unknown sources of thermal flows, etc. Experimental results for airborne transmission of aerosols are needed. Possible measurement techniques may be flow visualization as has been done by Verma *et al.*^12^ investigating face masks, or local measurement of particle concentration using particle collectors by Kinahan *et al.*^30^ in aircrafts, or particle size spectrometers by Zhang *et al.*^25^ in a bus, Zhang and Chen^31^ in a ventilated room or He *et al.*^32^ for wind-instruments.

As mentioned earlier, ventilation is regarded as a key mechanism to control aerosol and droplet spreading in indoor space. As pointed out, numerous studies investigated this aspect in the past using numerical, experimental, or combined methods. However, the presence of recirculatory flows driven by ventilation systems may not be sufficient to prevent a critical exposure of virus-laden droplet flows to the people in the room. Therefore, various air purifier systems have been developed in the times of the pandemic, which should help reduce the number of airborne aerosols in classrooms. Especially, if there are no windows available in the classroom, the ventilation with fresh air in cold seasons is not the method of choice.

Air purifier systems are used in several applications. Zhao *et al.*^33^ give a survey for the 100 best-selling systems comparing energy efficiency related to the reduction of the particle emission. Mousavi *et al.*^34^ examined the use of portable air purifiers and identified the best position of the purifier in hospitals by experiments. They found the best position close to the bed of the patient. An extensive experimental study was performed by Bluyssen *et al.*^35^ taking into account the aerosol transport and sound and air velocities in a SenseLab.

**FIG. 1.**
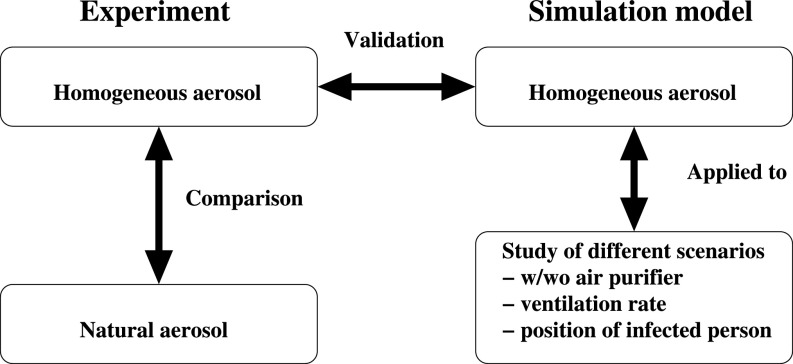
Graphical presentation of the workflow of experiments and numerical simulations.

The purpose of this study is to evaluate the efficiency of air purifier systems in classrooms under realistic conditions, i.e., including thermally driven flow effects. A numerical model is developed to describe the aerosol transmission. The model is validated with experimental results obtained in a classroom for a homogeneous distribution of an artificially generated aerosol. In a preliminary step, the natural aerosols in a classroom were measured and compared to the artificial aerosol to ensure comparable particle sizes. The validated simulation model is used to conduct different scenarios for different ventilation rates and locations of infected persons. The described workflow is shown graphically in Fig. 1.

The experimental conditions and setup are presented in Sec. II, followed by the presentation of the numerical model in Sec. III. The numerical results—validation and study of different scenarios—are summarized in Sec. IV, which is followed by a conclusion and outlook on further studies.

## II. SITUATION AND EXPERIMENTAL CONDITION

**FIG. 2.**
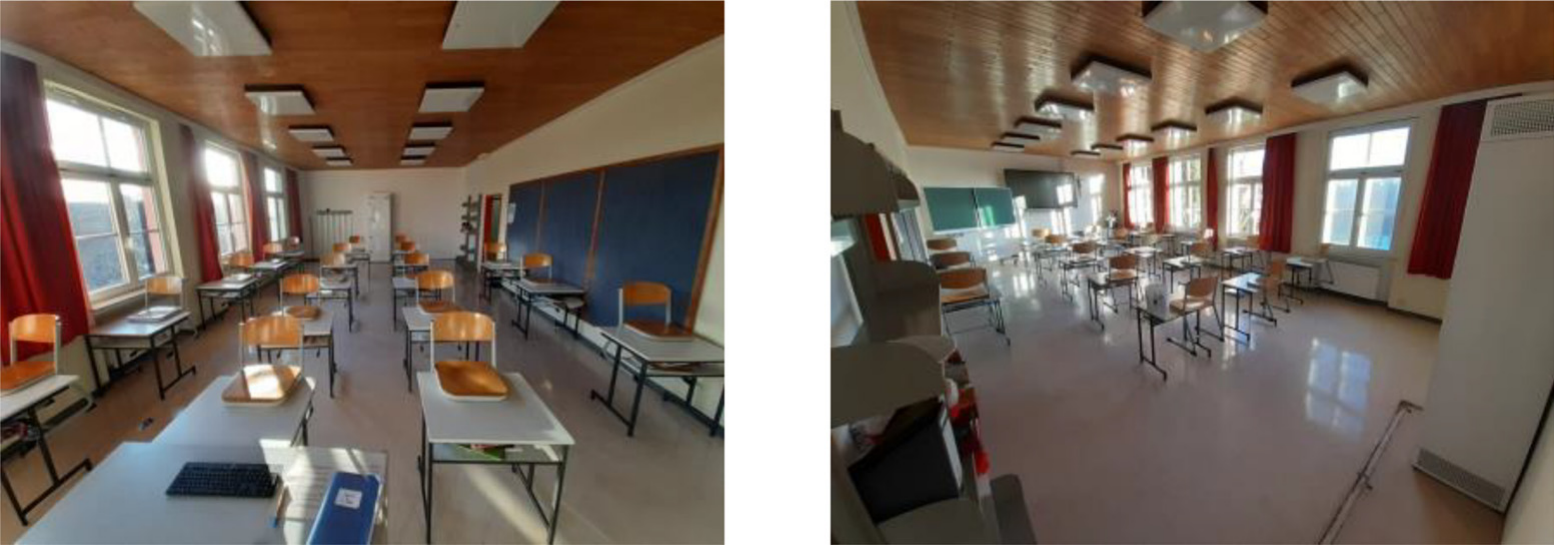
Photograph of the classroom (left: teachers' view; right: back of the room with air purifier).

For a realistic situation, a typical classroom of a school in Rohr (Bavaria, Germany) was chosen, which can be seen in Fig. 2. The dimensions of the room are 11.17 m × 5.7 m × 3.1 m. In the room, there are 18 students. The room is equipped with an air purifier at the back of the room. This specific air purifier is a stand-alone system, which is not connected to an air handler unit or HVAC unit. It is a passive system which cleans the air by a combination of carbon filter and high-efficiency particulate air filters (HEPA). The HEPA-H14 filter that is classified according to DIN EN 1822 filter removes 99.995% of particles. CADR (clean air delivery rate) is up to 1200 m^3^/h, which corresponds to the ventilation rate (VR) of 6/h. The ventilation rate in this study is defined as the amount of volume flow per hour filtered by the air purifier related to the volume of the classroom. Maximum power consumption is 280 W. The air purifier possesses an inlet of the air at the bottom on the left and on the right side. Cleaned air is injected in the room on the left and on the right side as well as on the front of the purifier 2.3 m above ground. In the building, there is no additional air-conditioning or ventilation system installed.

### A. Experimental setup

**FIG. 3.**
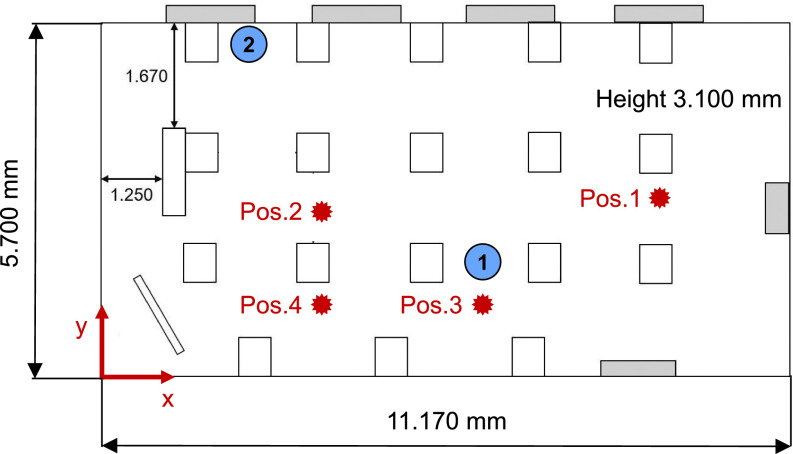
Dimensions of the classroom and the positions for the measurement of the aerosol concentration. The height of the measuring probes is 1.2 m. The circles in blue mark are the positions 1 and 2 where infected persons are located in the simulations.

Experiments are performed in the classroom on a usual school day to validate the numerical model and to model the classroom-situation as realistic as possible. The aerosol concentration is measured successively at 4 selected locations within the classroom (compare Fig. 3), and the temporal evolution of the particle number is monitored over time. A particle spectrometer (Optical particle sizer, OPS, Model 3300, TSI Incorporated) was used, which allows for an online measurement of aerosols providing a sample flow rate of 1 l/min. Additionally, a concentric 1 l/min of sheath flow is used to focus the particle beam. As an optical particle spectrometer, the OPS uses a laser-diode with 30 mW that emits light of 660 nm wavelength. The scattered light of the particles is detected by a photomultiplier. The detected light signal is related to the particle size. This particular aerosol spectrometer is capable of detecting particles from 0.3 to 10 *μ*m in diameter in up to 16 channels. The OPS is calibrated with polystyrene latex (PSL) particles. In case the measured aerosol has significant different refractive index, the OPS allows for real-time Mie scattering calculation to adjust the PSL calibration curve to a curve that better fits the aerosols of interest. Measurement accuracy concerning size resolution is stated to be 5% at 5 *μ*m according to the manufacturer. Further details can be found in Han *et al.*^36^ The virus carrying aerosol size is up to 5 *μ*m (Zhang *et al.*^25^); therefore, the OPS is suitable to measure the relevant particle concentration in a classroom. Sampling height for all measurement locations is at 1.2 m, i.e., approximately at height of the head of a sitting pupil. The coordinates of all 4 sampling points are shown in Table I.

**TABLE I.** Positions for the measurement of the aerosol concentration.

Probe #	Position x (mm)	Position y (mm)	Position z (mm)
1	8366	3200	1200
2	3166	3000	1200
3	5666	1500	1200
4	3166	1500	1200

Additional to the measurement of the natural aerosol distribution in typical lessons on a usual school day, in a second step an artificial aerosol is introduced into the classroom and the temporal evolution of the particle concentration is measured under this more defined condition. Due to the fact that the movement of pupils, opening and closing doors and windows, the use of overhead projector screen during the lesson, etc. lead to a natural undefined aerosol creation; a more defined initial aerosol concentration is needed to analyze the effect of an air purifier system.

**FIG. 4.**
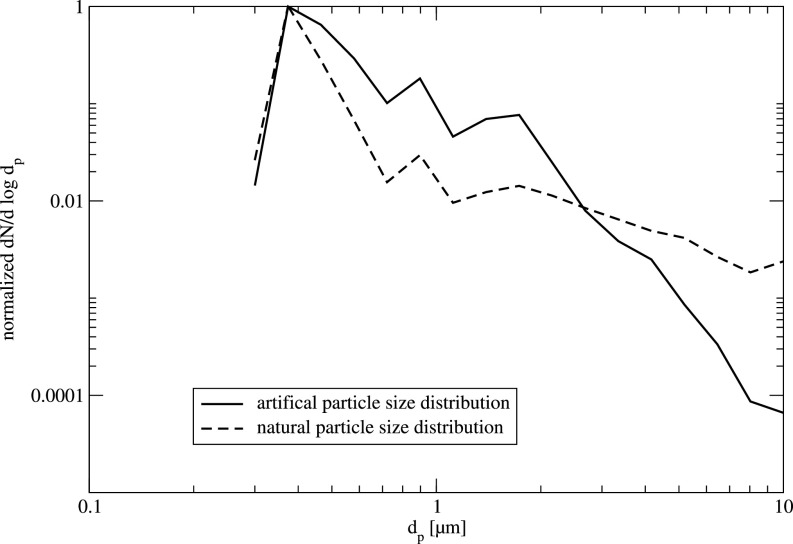
Typical normalized particle concentration for the natural and the artificial aerosol, normalized with maximum particle number.

An atomizer is used, which is equipped with a two-component injector nozzle. The fluid, which is DEHS (di-ethyl hexyl sebacate), is atomized using ambient air and provides particles with a diameter of mostly less than 2.5 *μ*m (compare Fig. 4). The aerosol is, therefore, sufficient for representing the natural virus-carrying aerosols in this study. Before each measurement, the empty classroom is seeded with DEHS aerosol for 5 min. Measurements of the particle concentration are performed at the same location as for the real environment in typical lesson but in an empty classroom now. After the room is sufficiently seeded, the air purifier is started and the particle concentration is measured using the OPS for a period of at least 30 min. In each case, a baseline-measurement is performed, i.e., the temporal evolution of the aerosol concentration is measured without operating the air purifier and without any other ventilation.

Ambient temperature in the classroom is between 20 °C and 25 °C at a relative humidity of 24% to 33%.

As mentioned earlier, in a first step, experimental investigations are performed, i.e., a local measurement of the aerosol concentration in the classroom is done for selected configurations with and without air purifier system. Experimental data already give insight into the effectiveness of the air purifier system.

### B. Experimental results

In the following, the experimental results are presented. These results will serve to validate the numerical results (see Sec. IV). As mentioned earlier, in the initial step of this study, the natural aerosol concentration in a classroom during lesson is measured. Note, in each lesson, the air purifier is operated continuously with 1200 m^3^/h. In a second step, an artificial aerosol is introduced in an empty classroom, and the particle concentration is measured when the air purifier is running. As can be seen in Fig. 4, the particle concentration distribution for the natural and the artificial aerosol is quite similar. The artificial aerosol has less particles with a diameter larger than 2.5 *μ*m. Nevertheless, this aerosol is suitable to mimic the natural particle size distribution as well as to investigate the temporal evolution of the critical particle sizes potentially carrying a virus.

**FIG. 5.**
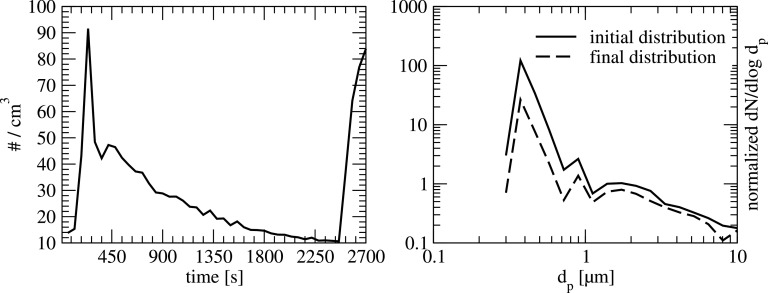
Left: typical temporal evolution of the detected total particle number at position 4 in a typical school lesson; doors are opened and pupils are moving at the beginning and at the end of the lesson. Right: corresponding particle size distribution at the beginning (t = 300 s) and at the end (t = 2400 s) of the air purifier operation-period for the natural aerosol in the classroom.

In the following, the results of the measurement under real conditions in the classroom are presented. A typical example of the temporal evolution of the particle concentration over time is shown in Fig. 5 (left) for the measurement position 4. In this figure, the total particle number for all 16 channels of the OPS is depicted.

In this particular case, an increase in particle number can be seen at the beginning of the lesson and at the end. The increase in particle number can be directly related to the movement of pupils and the opening of the door at the beginning and end of the lesson. Additionally, a decrease in the particle number can be clearly detected over a period of approximately 2000 s. Note, during the lesson, the air purifier is constantly running with 1200 m^3^/h. In between t = 300 s and t = 2400 s, there was no opening or closing of doors or windows, i.e., no artificial ventilation besides the air purifier-flow. A reduction of approximately 80% can be found within this period for particles smaller than 1 *μ*m as can be seen in Fig. 5 (right).

To get a clearer insight into the supposed effect of the air purifier, additional measurements using artificial aerosol in an empty classroom are performed. This artificial aerosol provides a higher number of particles such that statistical analysis can be done.

When an aerosol is introduced into a room by locally operating an atomizer, the generated particles will distribute within the room by natural convection. This settling time was analyzed performing baseline-experiments at each measurement location, i.e., introducing the aerosol and not performing any mean of ventilation.

At the beginning, the detected particle number is relatively high. But within a certain time, the aerosol has sufficiently distributed such that a constant particle number can be detected for the rest of the measurement time. Since there is a plateau of particle number over time, it can be concluded that sedimentation or evaporation does not lead to significant aerosol-decay for the ambient condition in the classroom.

After settling time was evaluated, the second experimental campaign is performed, i.e., the artificial aerosol is introduced and the air purifier is started. The particle concentration and its decay are measured up to 1800 s. The particle concentration significantly decreases over the measurement period (see Fig. 11 in Sec. IV). Pos. 2 to 4 show similar trends as they are at similar distance from the air purifier. At Pos. 1, which is at the closest distance from the air purifier, the decay of particle concentration has a steeper slope at the beginning. This can be associated with the locally stronger flow velocity induced by the air purifier (compare Sec. III), such that particle-laden flow is filtered faster.

**FIG. 6.**
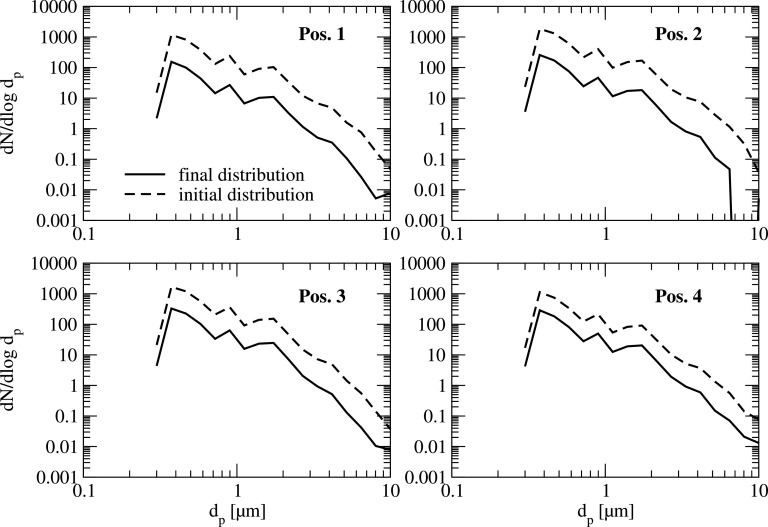
Typical particle size distribution at the beginning and at the end of the air purifier operation-period for the artificial aerosol in the classroom at all 4 measurement positions.

**FIG. 7.**
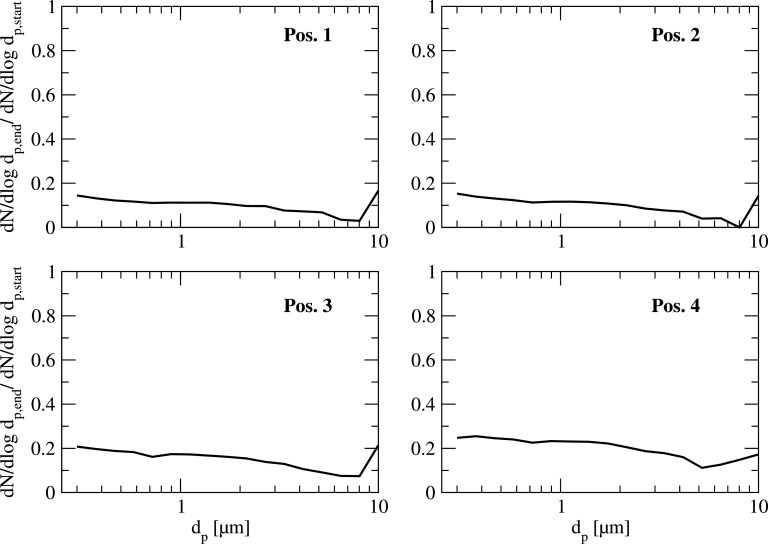
Typical reduction of particle concentration for the artificial particle size distribution with air purifier operation in the classroom at all 4 measurement positions.

A deeper insight into the measured decay for the complete size spectrum is illustrated in Figs. 6 and 7 for all measurement positions. In these figures, the normalized particle size distribution dN/dlogd_p_ is shown at the beginning and at the end of the measurement period (Fig. 6), and the ratio of these two values (Fig. 7) for each position. As can be seen, the decay of the particle concentration is almost uniform for the complete particle size range. Since the overall particle number for the artificial aerosol is much higher than for the natural aerosol even for larger particle sizes, statistical analysis can be done.

As can be seen in Fig. 6, the overall decay of the particle number is in the range of 78% to 83% within a time period of 28 min for all 4 measurement points. Although, at position 1, which is close to the air purifier, the decay of particle concentration is stronger in the initial phase (see Fig. 11), the overall reduction within the mentioned period is quite similar to the positions further away from the purifier (pos. 2 to 4).

Summing up, this experimental investigation shows that in this particular case, an air purifier system may lead to a reduction of critical particle sizes (less than 5 *μ*m) of at least 80% within 30 min. This conclusion can be drawn based on measurements of the natural aerosol concentration in a typical school lesson and measurements of artificial aerosol concentration.

The results are used in the following for a validation of a numerical simulation of the flow and particle distribution within a classroom.

## III. NUMERICAL ANALYSIS

In the numerical simulation, the distribution of the aerosol during one school lesson (45 min) should be studied. The modeling of the transient process that is combined with a change of the temperatures due to heating effects caused by persons or lamps, monitors, etc. requires some assumptions and simulation strategies to reduce the simulation times. The assumptions, the equations, and the simulation method used are stated in Secs. III A and III B.

### A. Simulation model and strategy

The flow inside the classroom is assumed to be incompressible and turbulent. The turbulent flow is modeled using the Reynolds-Averaged Navier-Stokes equations (RANS) with a k-Ω-SST (Shear Stress Transport) two-equation turbulence model. The conservation of mass and momentum using the Boussinesq approximation to account for a change of the density due to the temperature T can be written as

∇⋅u=0, (1)

$$\frac{\partial u}{\partial t}+\nabla \cdot \left(\mathrm{uu}\right)=-\nabla p-g\cdot x\;\nabla \left(\frac{\rho }{\rho_{0}}\right)+\nabla \cdot \left({\;\nu }_{\mathrm{eff}}\cdot \left(\nabla u+{\left(\nabla u\right)}^{T}\right)\right).$$ (2)

with the velocity vector **u**, kinematic pressure p, density of the gas ρ kand effective viscosity ν_eff_, which is the sum of the molecular and the turbulent viscosity. The reference density ρ_0_ is used for the Boussinesq approximation, which assumes a constant density except in the term for the gravity **g** to include buoyant effects due to temperature differences. Differences in the height are included with the spatial position **x**. The density ρ is derived with the thermal expansion coefficient β (β = 3 × 10^−3^ 1/K) and the reference temperature T_0_ (T_0_ = 300 K) as $\frac{\rho }{\rho_{0}}=1-\beta \left(T-T_{0}\right).$ The temperature T is calculated by solving a convection-diffusion equation with the diffusion coefficient α_eff_, which is the sum of the molecular and turbulent part $\alpha_{\mathrm{eff}}=\frac{\nu_{t}}{{Pr}_{t}\;}+\frac{\nu }{Pr}$ using the molecular and turbulent Prandtl number Pr = 0.7 and Pr_t_ = 0.85.

The turbulent properties for the turbulent viscosity are obtained by solving the transport equations for k and Ω in the k-Ω-SST RANS model

$$\frac{\partial T\;}{\partial t}+\nabla \cdot \left(uT\right)=\nabla \cdot \left(\alpha_{\mathrm{eff}}\cdot \nabla T\right).$$ (3)

Additionally, the CO_2_ concentration c_CO2_ is determined by an additional transport equation. The diffusional constant is assumed to be equal α_eff_ as the main part of the diffusion is invoked by turbulent diffusion

$$\frac{\partial c_{CO_{2}}}{\partial t}+\nabla \cdot \left(uc_{CO_{2}}\right)=\nabla \cdot \left(\alpha_{\mathrm{eff}}\cdot \left(\nabla c_{CO_{2}}\right)\right).$$ (4)

The distribution of aerosols as a particulate phase can be modeled either with an Lagrangian approach or based on a Eulerian approach. As the settling velocities of the COVID-19 aerosols, which are assumed to be smaller than 1 *μ*m, can be neglected, and therefore the particle relaxation time as well (Zhang *et al.*^25^), i.e., the aerosol is assumed to follow the flow. The distribution of the aerosol concentration c_Covid_ is described using the following equation:

$$\frac{\partial c_{\mathrm{Covid}}}{\partial t}+\nabla \cdot \left(uc_{\mathrm{Covid}}\right)=\nabla \cdot \left(\alpha_{\mathrm{eff}}\cdot \left(\nabla c_{\mathrm{Covid}}\right)\right).$$ (5)

With this approach, the transport of the aerosol of the infected person is described. An interaction and mixing of different aerosols exposed by infected and noninfected persons is neglected in this study.

All the equations are solved with the open source CFD-code OpenFOAM® (version 6), which is based on a Finite-Volume method. For the discretization of the temporal and spatial discretization, schemes of second order are used.

The coupled simulation of flow and aerosol transport requires a huge number of time steps to fulfill the stability criteria for a Courant number less than 1. This leads to long simulation times especially on fine meshes. Therefore, two modeling approaches have been considered in this study:

(1) Coupled simulation of flow and aerosol transport with the transient flow simulation (named **transient** in Table II).
(2) Calculation of the flow field based on the assumption of steady-state flow field, which is followed by a transient calculation of the aerosol transport based on the assumption of a frozen flow (named **frozen** in Table II).

**TABLE II.** Case setup used for the validation of the model and parameter studies for the infected persons.

Run #	Source of aerosol	Grid^a^	Modeling approach	Ventilation rate (1/h)
1	Homogeneous	Coarse	Frozen	6
2	Homogeneous	Medium	Frozen	6
3	Homogeneous	Fine	Frozen	6
4	Homogeneous	Coarse	Transient	6
5	One person, position 1	Coarse	Frozen	0
6	One person, position 1	Coarse	Frozen	3
7	One person, position 1	Coarse	Frozen	6
8	One person, position 1	Coarse	Frozen	12
9	One person, position 2	Coarse	Frozen	0
10	One person, position 2	Coarse	Frozen	6

^a^ The grid for the simulations with a homogenous concentration does not include persons.

Both methods have been evaluated and validated with the experimental results (see Sec. IV).

### B. Computational domain and boundary conditions

**FIG. 8.**
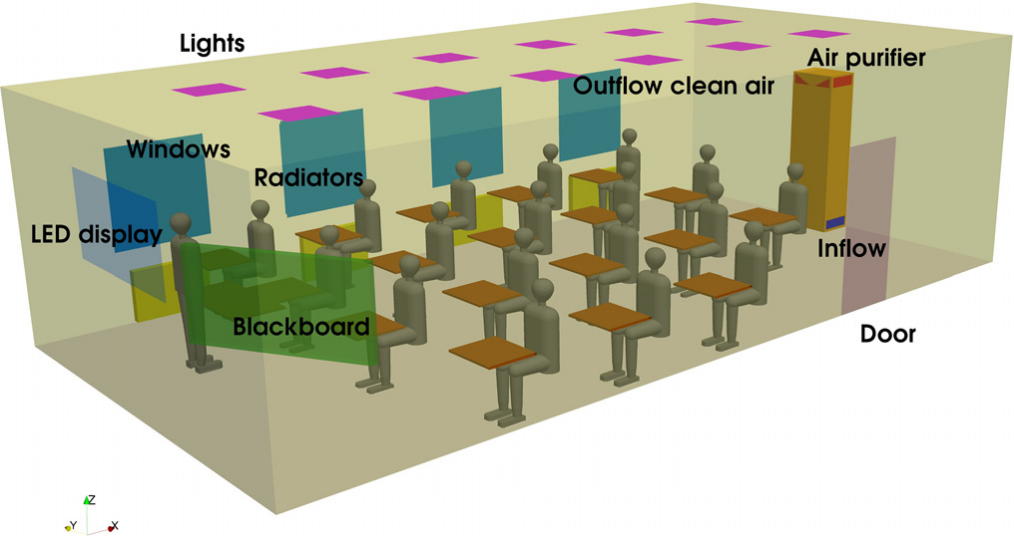
Configuration of the simulation model.

The geometry of the simulation model is shown in Fig. 8. In the room, there are 18 students and one teacher. All the relevant parts are included in the model in a simplified way. The air is cleaned by high-efficiency particulate air filters (HEPA) with the inlet of the air at the bottom on the left and on the right side of the air purifier. The cleaned air is injected in the room on the left and on the right side as well as on the front of the purifier. In a preliminary study, the flow conditions (distribution of flow rate and angle of jet) are determined in a detailed CFD study of the air purifier and included in the large model as a boundary condition for the velocity.

The air purifier has a total volume flow of 1200 m^3^/h, which is distributed on the left and right side (each 39%) and the front (22%). The angle on the right and left side is inclined 25° to the horizontal plane, which is validated by measurements. The jet at the front has an angle of 39° to the horizontal plane. At the outlet of the air purifier, a turbulence intensity of 2.5% and turbulence length scale 0.1 m are assumed. The concentration of the aerosols at the outlet is assumed to be zero, i.e., the air is cleaned to 100% from aerosols in the purifier. The concentration of CO_2_ and the temperature are transferred from the inlet to the outlet of the purifier. Each student and the teacher have a volume flow of 4 m^3^/h with a concentration of 5% CO_2_ and a temperature of 303 K. The infected person injects 50 aerosol particles each second.^18^ The turbulence intensity of 10% and the turbulence length scale of 7.5 mm are assumed to be at the mouth of each person. Each person heats the room with 50 W. At the outer walls of the room, a heat transfer coefficient (HTC) of 2 W/m^2^ K and an ambient temperature of 278 K for the calculation of the heat flux are assumed. The heat transfer coefficient (HTC) was obtained by a preliminary numerical study (not reported here) with different values for the HTC and a comparison with measurements of the temperature in the classroom. The temperature of the lamps and LEDs is assumed to be 298 K, whereas the heating has constant values of 303 K. The values used in the simulation are mean values obtained by measurements with a thermal camera during the experiments.

### C. Parameter studies

To study the transport of SARS-CoV-2 (COVID-19) aerosols, different cases have been examined, which are listed in Table II. The experiments with a homogenous distribution of aerosols at the beginning were used for the validation of the numerical model, including a grid dependency study (runs 1–3). To verify the difference of a fully coupled simulation vs frozen flow simulation, a transient simulation was considered in run 4. The validated numerical model is used to study the influence of the ventilation rate with an infected person at position 1 (runs 5–8), the influence of the different position 2 of the infected person (runs 9 and 10), and the influence, if no air purifier is used (runs 5 and 9). The positions 1 and 2 can be seen in Fig. 3.

## IV. RESULTS AND DISCUSSION

The results of the numerical simulations are discussed in Secs. IV A–IV C.

### A. Grid refinement study

**FIG. 9.**
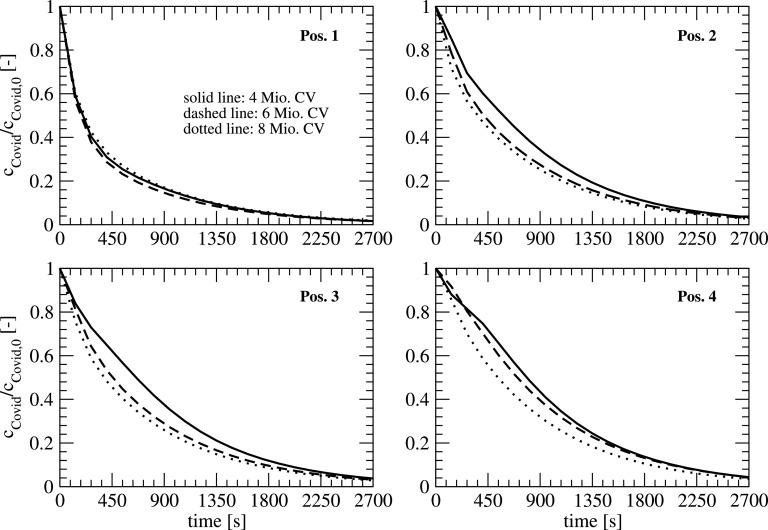
Temporal development of the concentrations at different locations for different mesh resolutions.

To make sure that the results are grid independent, a grid refinement study was performed with three different mesh sizes using 4.33, 5.93, and 8.25 × 10^6^ control volumina (CV) for the geometry including persons. The grid was generated based on the geometry in Fig. 3 using the automized meshing tool snappyHexMesh from the OpenFOAM® toolbox. The different mesh sizes are obtained by a refinement of the background mesh. The background mesh is a hexahedral mesh, which is snapped and refined at boundaries with 8 boundary layers. The mean grid resolution in the flow domain is approximately 100 mm, 80 mm, and 65 mm. A comparison of the transient behavior of the aerosol concentration at the locations 1–4 (see Fig. 3), which is made dimensionless with the initial concentration for three different meshes (runs 1–3), is plotted in Fig. 9.

All the positions show a similar decay of the concentration. The differences between the mesh sizes of 6 and 8 Mio. are quite small, except for position 4 where a slight difference can be observed. The correlation between the local convergence and overall number of control volumina is not linear as the local mesh density is not correlated linear with the overall number of the control volumina in the meshing process. As the differences are small, the coarse mesh is used for the following simulations.

**FIG. 10.**
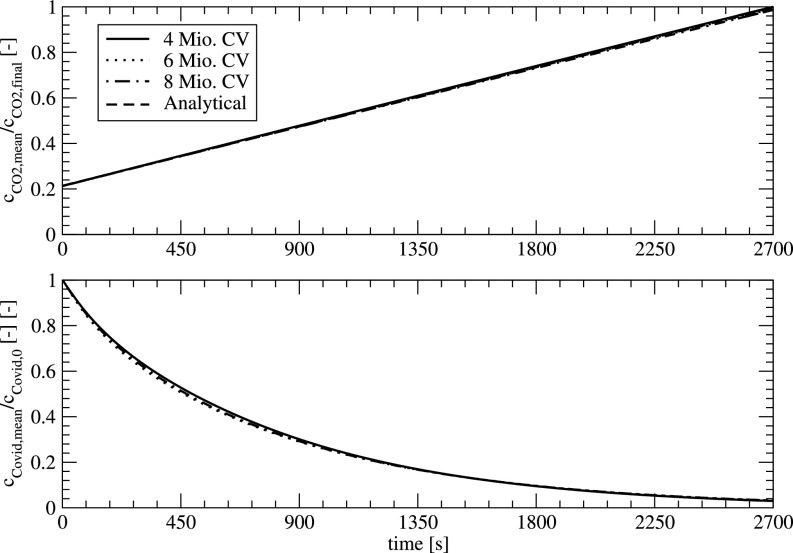
Temporal development of the CO_2_ content and COVID concentration for different mesh resolutions.

Figure 10 shows the dimensionless concentration of CO_2_ (related to the CO_2_ value for 2700 s) and the dimensionless COVID concentration (related to the initial concentration) in the classroom for the three different meshes. The CO_2_ values show an excellent agreement with the analytical value of CO_2_ proving the conservation of the scalar quantities.

### B. Validation of the numerical model

**FIG. 11.**
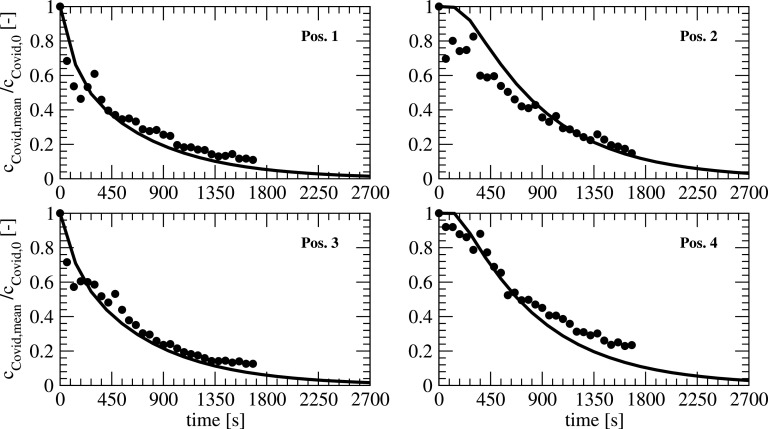
Temporal development of the concentrations at different locations for numerical and experimental results.

For the validation of the model, the experiments based on an initial homogenous concentration of aerosols are used as a reference. Figure 11 shows the time history of the dimensionless concentrations for the positions 1–4. Based on the grid refinement study, the numerical results are based on simulations with a coarse mesh. As a reference, the values in the experiment are normalized after 120 s of handling time to start the experiment after a homogenous concentration in the room is established. For the comparison of experiment and numerical results, all the persons in the simulation model have been removed to have identical conditions like in the experiment. The comparison between all the positions shows a good agreement. On position 2, the fluctuations in the experiments are higher for smaller times. Due to the experimental setup, a development of the flow is included in the experiment, whereas in the flow simulations, a developed flow is used as a starting point.

**FIG. 12.**
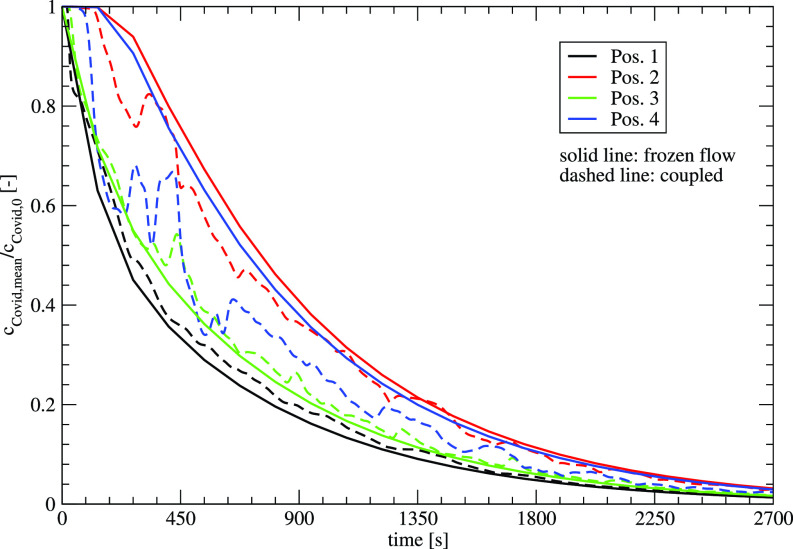
Temporal development of the concentrations at different locations for the two numerical approaches—frozen flow and coupled flow simulation.

Figure 12 shows the concentrations for the two numerical approaches—frozen flow and coupled—as a function of time. Positions 1 and 3 that are close to the air purifier show slight differences between the two approaches. For a further distance at positions 2 and 4, the fluctuations of the flow can be observed with smaller differences at position 2 and larger differences for position 4. For larger time scales, all the positions show similar behavior for the two numerical methods, which allow the use of frozen flows for the prediction of larger time scales. If the transient behavior, especially at the beginning, has to be considered, the coupled approach is mandatory.

### C. Aerosol distribution for different scenarios

With the validated model, three different scenarios are considered:

• Influence of ventilation rate
• Influence of different positions of infected persons
• Influence, if no air purifier is working

#### 1. Influence of ventilation rate

**FIG. 13.**
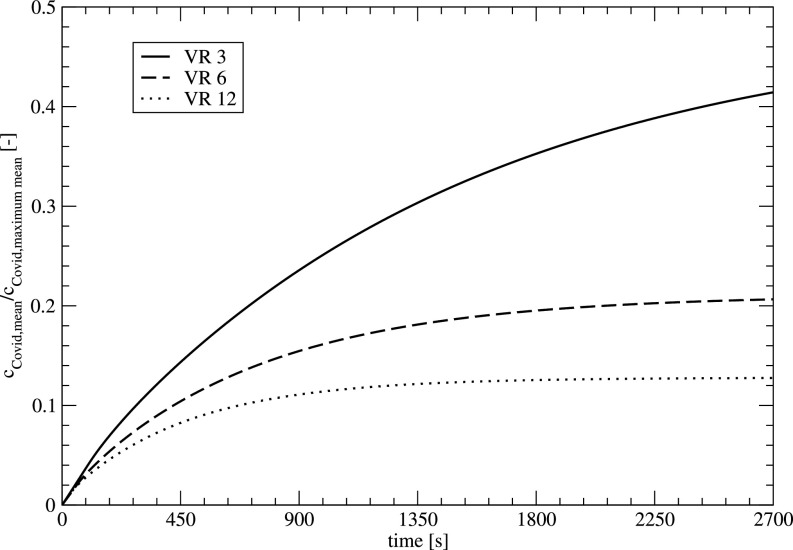
Temporal development of the mean aerosol concentration for different ventilation rates, infected person at position 1.

Figure 13 shows the development of the mean aerosol concentration in the room for different ventilation rates. The infected person is sitting at position 1. The influence of the ventilation rate (VR) shows the expected behavior with a rapid reduction of the mean concentration for higher VRs. For higher VR, a nearly constant concentration for higher times can be observed, where aerosols are located in regions with small velocities where only diffusional effects on a larger timescale lead to a reduction of the aerosol.

**FIG. 14.**
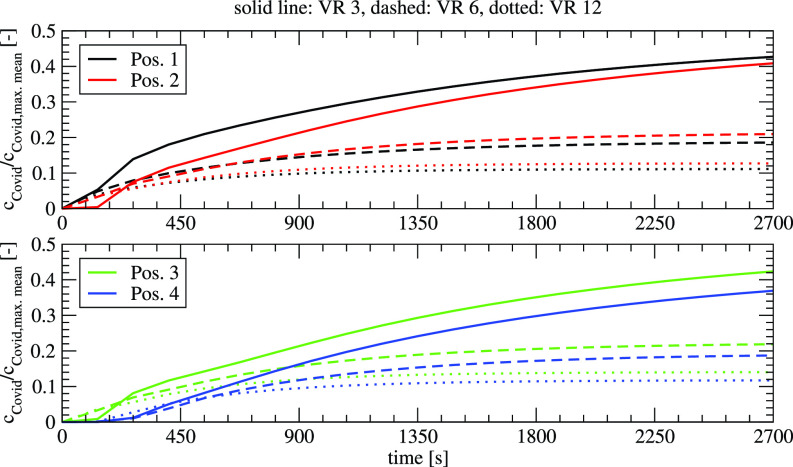
Temporal development of the local concentration for different ventilation rates, infected person at position 1.

Figure 14 shows the development of the aerosol concentration at the locations 1–4 for the measurement of the three different VRs. Like the mean concentrations, the concentrations at different locations decrease with higher VR.

**FIG. 15.**
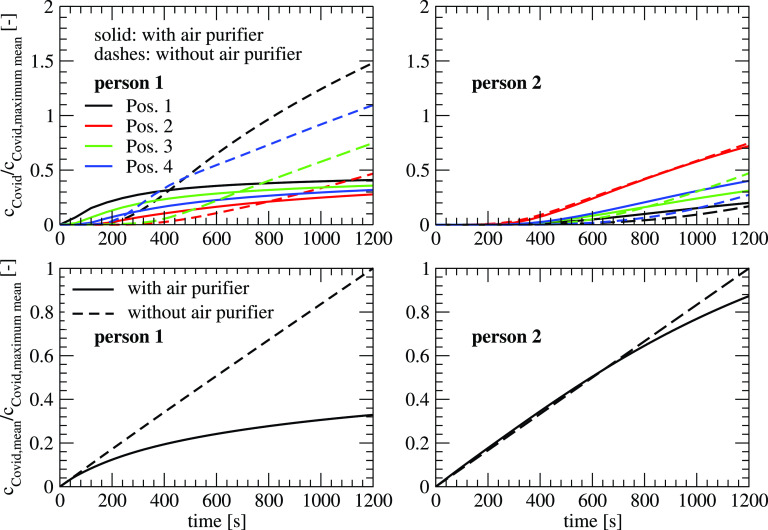
Temporal development of the concentration at different positions (top) and mean concentration (bottom) with and without an air purifier (left: infected person at position 1; right: infected person at position 2).

Figure 15 shows the influence of the air purifier (VR 6) on the concentration at different locations for the two cases of infected persons. If the infected person is at location 1, the use of the air purifier leads to a faster distribution of the aerosol at the different locations. For the case without the air purifier, the transport that is dominated by diffusion is slower leading to smaller concentrations up to a time between 400 s (position 1) and 1100 s (position 2). After these times, the air purifier reduces the load on aerosols efficiently. If the infected person is located at position 2 which has a larger distance to the locations 1–4, it takes 200–500 s till an increase in the concentration can be detected. The difference between the values with and without air purifier is relatively small as the convective transport at location 2 of the infected person is relatively small. If one compares the mean concentration in the room, the mean concentration is reduced to 32% if the infected person is at location 1, and 88% at location 2. This shows the importance of the placement of the air purifier in the room, which can influence the efficiency of the system dramatically.

## V. CONCLUSIONS

In this paper, the transmission of aerosols in a classroom is studied numerically and experimentally for different scenarios. The experiments are performed in a classroom using DEHS aerosol, which is measured with an aerosol spectrometer. The transient reduction of aerosol is determined at different locations with and without using an air purifier. The reduction ranges between 70% and 90% dependent on the locations if a ventilation of 6/h is applied.

The numerical model is able to reproduce the experimental results with a good accuracy. With the validated numerical model, different scenarios have been analyzed. An increase in the ventilation rate reduces the aerosol. It can also be shown that the position of the infected person influences the concentration in the room significantly. Dependent on the position (i.e., for larger distances between the infected person and the air purifier), the air purifier may lead to a higher load of concentrations at different locations of the measurement.

With the proven reduction of potentially virus-laden aerosols, the air purifier is a useful addition to window ventilation. For further product generations, an analysis of the effect on the flow distribution can improve the performance of the air purifier systems. Especially, the influence of the infected person, position of the air purifier, and the outflow characteristics are currently considered in an optimization study, which should give detailed recommendations for the placement of the air purifier in the room.

## Data Availability

The data that support the findings of this study are available from the corresponding author upon reasonable request.
